# New Appliances and Things Medical

**Published:** 1899-03-18

**Authors:** 


### NEW APPLIANCES AND THINGS MEDICAL.

**[We shall be glad to receive, at our Office, 28 & 29, Southampton Street, Strand, London, W.C., from the manufacturers, specimens of all new preparations and appliances which may be brought out from time to time.]**

### EXONIA MALT PREPARATIONS.

(Evans, Gadd, and Co., Fore Street, Exeter.)

Exonia Malt Extract is a preparation of high diastatic power and of pleasant flavour. It is dispensed in an elegant form in convenient bottles with patent caps, which ensure cleanliness, economy, and accuracy of dose. Ambrosia is a combination of the above malt extract with rich pure cream. The manufacturers claim credit for devising a special apparatus for combining the two ingredients and for exercising the greatest care in obtaining the cream by a mechanical separator, and subsequently sterilisation by the most approved method. Ambrosia is exceedingly pleasant and is much liked by children. Ambrosia pastilles demonstrate an ingenious extension of our present methods of administering useful medicaments in pleasant form. They dissolve rapidly in the mouth, are apparently absolutely harmless even when taken in considerable quantities, and they certainly have the power of relieving dryness of the mouth and huskiness of the voice. They should be useful for public vocalists and speakers.

### KRONTHAL WATERS.

(Importers, Schweppe's, Limited, 53, Berners Street, W.)

These waters, which are bottled at the springs in Kronthal, in the Prussian province of Hesse-Nassau, are included under the group of muriated alkaline waters. They are being largely imported by Messrs. Schweppe, and are distinguished by three different labels, blue, red, and green. The blue label belongs to a natural sparkling table water, agreeable to the taste, and suitable for combination with wines and spirits. This water is, perhaps, chiefly indicated in those cases where there is a tendency to the formation of uric acid calculi and gravel; it is also suitable for patients suffering from chronic catarrh of the respiratory organs. The red label is a more highly mineralised sparkling water; it also may be used for table purposes, and is highly agreeable. Its chief application is for more pronounced cases of the uric acid diathesis. As an eliminant of the acid from the blood it has a high reputation. The green label is a sparkling iron water, in which the iron exists in valuable form for those of weak digestion. It can be readily assimilated, and should be specially recommended in cases of anæmia combined with dyspeptic symptoms.

### AUSTRALIAN BURGUNDY (HARVEST BURGUNDY AND TINTARA).

(P. B. Burgoyne and Co., 5, Dowgate Hill, London, E.C.)

The sample of Harvest Burgundy (Vineyard Brand Red) which has been forwarded us for examination is a fine natural Australian wine of excellent quality. Though a wine of considerable strength and alcoholic percentage, it is noticeably free from the crude roughness which makes many Australian wines unpalatable to the English taste. It is a wine which in our own experience has been followed by the most pleasing results in cases of post-influenza debility. It makes an excellent mulled beverage, and as a rule we would recommend it well diluted. Tintara is an excellent dry wine of full body and of ferruginous properties. It is admirably adapted for anæmic persons, or those who require a wine with the strengthening properties of a port without the disadvantages of a large excess of sugar.

